# Placenta-Specific Protein 1 Is Conserved throughout the Placentalia under Purifying Selection

**DOI:** 10.1155/2014/537356

**Published:** 2014-08-07

**Authors:** Eric J. Devor

**Affiliations:** Department of Obstetrics and Gynecology, University of Iowa Carver College of Medicine, 3234 MERF, 375 Newton Road, Iowa City, IA 52242, USA

## Abstract

Placental mammals (Placentalia) are a very successful group that, today, comprise 94% of all mammalian species. Recent phylogenetic analyses, coupled with new, quite complete fossils, suggest that the crown orders were all established rapidly from a common ancestor just after the Cretaceous/Tertiary (K/T) boundary 65 million years ago. Extensive molecular and morphologic evidence has led to a description of the common ancestor of all Placentalia in which a two-horned uterus and a hemochorial placenta are present. Thus, the process of placentation in which the placenta invades and anchors to the uterine epithelium was already established. One factor that has been suggested as a crucial component of this process is placenta-specific protein 1 (PLAC1). A phylogenetic analysis of the PLAC1 protein in 25 placental mammal species, representing nine of the sixteen crown orders of the Placentalia, suggests that this protein was present in the placental common ancestor in the form we see it today, that it evolved in the Placentalia and has been subject to the effects of purifying selection since its appearance.

## 1. Introduction

Placenta-specific protein 1 (PLAC1) is a small (212-amino acid) secreted protein encoded by a gene located in the human genome at Xq26 near the HPRT locus [1]. Expression of PLAC1 in normal cells is limited to placental trophoblasts [2]. To date, PLAC1 is unique among genes either predominantly or exclusively expressed in the placenta in the fact that it does not contain placenta-specific promoters and it is not a member of a multigene family nor did it originate from an endogenous retrovirus [3]. The mature PLAC1 protein consists of a transmembrane domain and an extracellular domain containing a region homologous to the N-terminal subdomain of the zona pellucida ZP3 glycoprotein [4, 5]. The ZP3-like region classifies PLAC1 as a cell adhesion molecule and hints that strong protein binding interactions are possible [6] leading to the suggestion that it is involved in trophoblast invasion of the uterine wall and anchoring of the placenta to the endometrium [2].

The creation of a PLAC1 knockout mouse revealed that loss of PLAC1 results in placentomegaly and intrauterine growth restriction (IUGR) leading to the conclusion that PLAC1 is essential for normal placental and embryonic development [7]. Given the apparent importance of PLAC1 for placentation, a phylogenetic analysis of PLAC1 in 25 placental mammal species representing more than half of the mammalian crown orders is reported. Results suggest that PLAC1 is likely present in all placental species and has been under significant purifying selection throughout the history of the clade.

## 2. Materials and Methods

Using the PLAC1 cDNA sequence from human PLAC1 mRNA (NM_021796) as the starting point, 42 mammalian genomes in ENSEMBL were screened via BLAST. For this round, default parameters were employed and only local sequence dissimilarities were accepted. Among these, the PLAC1 coding region could be unambiguously identified in a total of 25 species representing nine crown orders of the Placentalia. Crown orders are Primates, represented by nine species (human,* Homo sapiens *(Has); chimpanzee,* Pan troglodytes* (Ptr); gorilla,* Gorilla gorilla* (Ggo); orangutan,* Pongo abelii* (Pab); gibbon,* Nomascus leucogenys* (Nle); rhesus macaque,* Macaca mulatta* (Mml); baboon,* Papio hamadryas* (Pha); marmoset,* Callithrix jacchus *(Cja); and galago,* Otolemur garnettii* (Oga)); Rodentia, represented by four species (mouse,* Mus musculus* (Mmu); rat,* Rattus norvegicus* (Rno); squirrel,* Ictidomys tridecemlineatus* (Itr); and kangaroo rat,* Dipodomys ordii* (Dor)); Carnivora, represented by four species (dog,* Canis familiaris* (Cfa); cat,* Felis catus* (Fca); panda,* Ailuropoda melanoleuca* (Ame); and ferret,* Mustela putorius* (Mpu)); Artiodactyla, represented by three species (cow,* Bos Taurus* (Bta); pig,* Sus scrofa* (Ssc); and Dolphin,* Tursiops truncatus* (Ttr)); Perissodactyla, represented by horse,* Equus caballus *(Eca); Afrotheria, represented by elephant,* Loxodonta africana* (Laf); Chiroptera, represented by the microbat,* Myotis lucifugus* (Mlu); Lagomorpha, represented by rabbit,* Oryctolagus cuniculus* (Ocu); and Scandentia, represented by the tree shrew,* Tupaia belangeri* (Tbe).

The 25 PLAC1 coding region DNA sequences were aligned using MUSCLE (v3.7) [8]. The alignment was curated with Gblocks (v0.91b) [9] and a phylogenetic tree constructed using the maximum likelihood method in PhyML (v3.0 aLRT) [10, 11]. Support for the tree was determined by bootstrap and the tree rendered in TreeDyn (v1983) [12]. All of the above analyses were implemented in* Phylogeny.fr* using default parameters [13].

Amino acid translations from the 25 coding region DNA sequences were carried out and used in tandem with the DNA sequences to estimate selection. Selection on both intact PLAC1 protein and PLAC1 functional domains, represented as dN/dS, was determined for species pairs using PAL2NAL [14] in which the dN/dS ratio is computed from the synonymous (dS) and nonsynonymous (dN) substitution rates calculated by codeml in PAML [15].

## 3. Results

**Figure 1:**
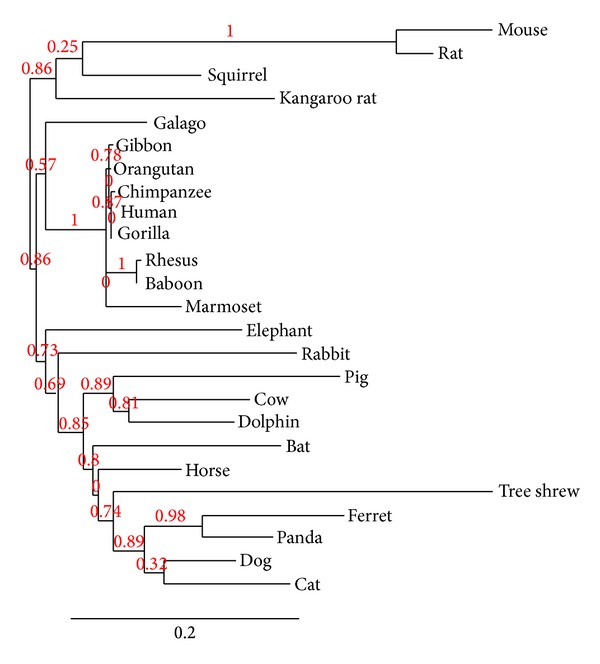
Best fit PLAC1 phylogeny among placental mammals based upon the coding region sequence. Maximum likelihood bootstrap support values are shown on nodes.

As noted, a total of 42 mammalian genomes were screened via BLAST in ENSEMBL using the human PLAC1 cDNA sequence as a starting point. Among these 42 species were 25 in which the PLAC1 cDNA could be unambiguously identified. In most of the remaining 17 genomes partial matches were seen but the sequences were incomplete with some or most of the coding region missing. In these cases, searches were carried out in the NCBI Trace Archive but without success. For the 25 species that did yield intact PLAC1 coding region sequences a phylogenetic analysis was performed. The resulting phylogenetic tree is presented in Figure 1. The phylogenetic relationships represented are for the most part orderly and conform to those produced using much larger data sets [16]. All four of the crown orders represented here by more than one species, Primates, Rodentia, Carnivora, and Artiodactyla, are grouped with substantial bootstrap support. Among the four crown orders represented by only a single species, Afrotheria (*Loxodonta africana*), Lagomorpha (*Oryctolagus cuniculus*), Chiroptera (*Myotis lucifugus*), and Perissodactyla (*Equus caballus*), only the horse occupies a phylogenetic position that is not well supported. This may be due to the placement here of* Tupaia belangeri*, the representative of the Scandentia, nearer to the Carnivora than* Myotis lucifugus*, a placement that is not consistent with other studies [16]. Most analyses of the tree shrew place it near or even within the Primates [17].

**Figure 2:**
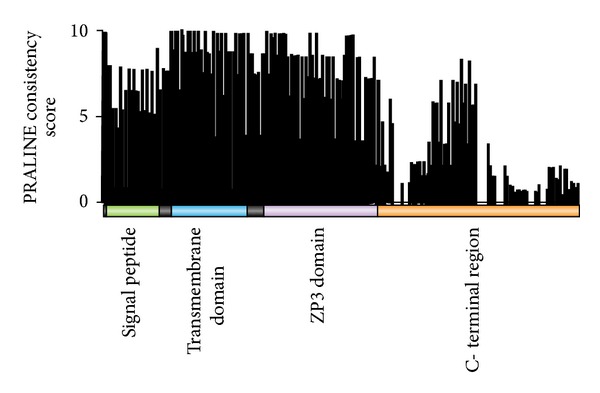
PLAC1 amino acid consistency scores by protein domain. A consistency score of 10 indicates no amino acid difference at the indicated position among all 25 placental mammal species.

PLAC 1 protein contains three functional domains. Using human PLAC1 amino acid sequence numbering (NP_068568), these are the signal peptide (residues 1–23), a transmembrane domain (residues 28–50), and a potentially highly reactive ZP3 domain (residues 58–118) [2, 7]. The remainder of the protein, residues 119-Cter, does not conform to any known conserved protein element. A PRALINE [18, 19] alignment of the 25 PLAC1 amino acid sequences showed that there is a high level of conservation over most of the protein. Using the 0 to 10 amino acid consistency score provided in PRALINE, conservation is highest in the 23 residues of the transmembrane domain and the 63 residues of the ZP3 domain, averaging 9.3 ± 1.2 in the former and 8.7 ± 1.2 in the latter (Figure 2). By comparison, the 23 residues of the signal peptide are less conserved at 7.0 ± 1.1 and the variable length C-terminal region is even less conserved at 4.0 ± 2.7. Much of the lack of conservation of the C-terminal region is due to a number of species having PLAC1 proteins that are truncated at the C-terminal end. As seen in Figure 2, a substantial drop in consistency score begins just a few positions after the ZP3 domain. However, there is a short region of 24 amino acids (residues 148–170) in which conservation is nearly as high as the signal peptide (6.7 ± 1.1). It is possible that this sequence serves an as yet unknown function but there is no extant evidence to support this idea. Among the 25 species in this study, 19 have a PLAC1 protein between 208 and 213 amino acids in length with the most common length being 212 amino acids. The remaining six, which include cow, pig, dolphin, mouse, rat, and bat, have PLAC1 proteins between 173 and 183 amino acids in length. All of the alterations in PLAC1 length in these six species are the result of shortened C-terminal domains. However, no shortened PLAC1 protein terminates before residue 170 which is the C-terminal-most amino acid of the 24 in the more conserved C-terminal region. It should be noted that the PLAC1 protein of the tree shrew, taken here to be the species most like the common ancestor of all Placentalia, is 211 amino acids long.

**Figure 3:**
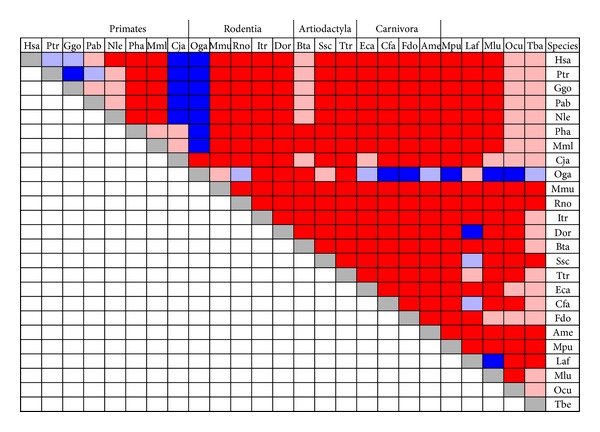
Pairwise dN/dS ratios among all 25 mammalian species in this study. Red represents 0.0 < dN/dS < 0.5. The pink entries represent 0.5 < dN/dS < 1.0; light blue represents 1.0 < dN/dS < 2.0; dark blue represents dN/dS > 2.0. Species abbreviations are given in the text. Crown orders containing more than a single representative are grouped together.

Estimates of selection on total PLAC1 in the form of the ratio of nonsynonymous (dN) and synonymous (dS) mutation rates, dN/dS, were computed using paired coding region DNA and protein amino acid sequences for all 300 possible species combinations. Results of these 300 pairwise species analyses are presented in graphical form in Figure 3. A dN/dS ratio of 1.0 is an indicator of neutral evolution whereas dN/dS > 1.0 is an indicator of diversifying selection and dN/dS < 1.0 is an indicator of purifying selection [20, 21]. As can be seen, no dN/dS ratios equal to 1.0 were obtained. Moreover, more than 90% (271 of 300) of estimates were less than 1.0 with 82.3% of these (223 of 271) less than 0.5. Of the dN/dS estimates greater than 1.0, more than half (16 of 29, 55.2%) were associated with comparisons involving galago (Oga) PLAC1 and an additional five were associated with comparisons involving the New World Monkey marmoset (*Callithrix jacchus*) and Old World Monkey and ape species. Overall, there appears to be sufficient evidence to suggest that PLAC1 has been subject to purifying selection throughout the Mammalia.

As it is well understood that selection can and does vary within genes as well as between species, dN/dS ratios were estimated for each of the three functional domains of the PLAC1 gene as well as the C-terminal region. Results for the Rodentia and the Carnivora are shown in Table 1. In both orders the signal peptide and the C-terminal domains exhibit weaker purifying selection but the transmembrane and reactive ZP3 domains exhibit strong purifying selection. Thus, the transmembrane and ZP3 domains would seem to be the two crucial components of PLAC1 but some level of conservation in the signal peptide and even in the C-terminal domain is in evidence.

## 4. Discussion

A phylogenetic analysis of placenta-specific protein 1 (PLAC1) in 25 placental mammal species representing nine crown orders is presented here. The results shown suggest that PLAC1 is highly conserved in the placental mammals. This is consistent with the conclusion from functional studies that PLAC1 is an essential component of normal placentation [2, 4, 7]. Some further support for the notion that PLAC1 is a key element in normal placental development and maintenance comes from the location of the PLAC1 gene in a region of the X-chromosome near HPRT, a region in which deletions are known to result in placental abnormalities [22].

 Indirect support for the idea that PLAC1 function is crucial for normal placental development and maintenance comes from the discovery that PLAC1 is expressed in a variety of human solid tumors including gastric cancers [23], non-small cell lung cancers [24], breast cancers [5], hepatocellular and colorectal cancers [25, 26], and endometrial and ovarian cancers [27, 28]. This pattern of expression led to the designation of PLAC1 as an “oncoplacental” protein, a class of proteins in which it remains the sole member [29]. Studies of PLAC1 expression in MCF7 and BT-549 breast cancer cell lines, including siRNA knockdowns, showed that PLAC1 expression is a vital component of cellular phenotypes including motility, migration, and invasion [5]. It is likely that PLAC1 carries out the same functions in the placenta and is switched on in solid tumors for this reason.

## 5. Conclusions

Placenta-specific protein 1 (PLAC1) is an essential component of normal placental development and maintenance. This is supported by both functional studies of PLAC1 and the phylogenetic analysis presented here. PLAC1 is likely ubiquitous in the Placentalia and is governed by significant purifying selection. There is evidence of PLAC1 sequence in a number of other placental mammal genomes in ENSEMBL though these genomes are not yet complete enough for the X-chromosome region in which the PLAC1 gene is located to be included here. The syntenic X-chromosome region of three marsupial species (wallaby,* Macropus eugenii*; Tasmanian devil,* Sarcophilus harrisii*, and opossum,* Monodelphis domestica*) and one monotreme (platypus,* Ornithorhynchus anatinus*) genome, focusing on the chromosomal region around the HPRT locus, has been systematically searched and no sequences similar to PLAC1 have been found. In addition, BLAST searches of these species using all or parts of the PLAC1 coding region and allowing very relaxed criteria failed to identify similar sequences in any other region of the marsupial or monotreme genomes. Though not conclusive, these results are consistent with a hypothesis that PLAC1 evolved with and exclusively in the Placentalia near HPRT and that it has been under purifying selection from its emergence as it carries out a crucial role, or roles, in placentation.

## Figures and Tables

**Table 1a:** (a) Rodentia

	Signal peptide				Transmembrane				ZP3				C-terminal			
	Mmu	Rno	Itr	Dor	Mmu	Rno	Itr	Dor	Mmu	Rno	Itr	Dor	Mmu	Rno	Itr	Dor
Mmu	—	0.24	0.38	0.47	—	0.11	0.01	0.00	—	0.11	0.08	0.09	—	0.13	0.61	0.66
Rno		—	0.45	0.55		—	0.00	0.00		—	0.09	0.10		—	0.08	0.80
Itr			—	0.32			—	0.00			—	0.07			—	0.82
Dor				—				—				—				—

**Table 1b:** (b) Carnivora

	Signal peptide				Transmembrane				ZP3				C-terminal			
	Cfa	Fdo	Ame	Mpu	Cfa	Fdo	Ame	Mpu	Cfa	Fdo	Ame	Mpu	Cfa	Fdo	Ame	Mpu
Cfa	—	0.33	0.89	1.17	—	0.23	0.00	0.00	—	0.15	0.07	0.07	—	0.82	0.38	0.51
Fdo		—	99.0	0.73		—	0.12	0.00		—	0.10	0.13		—	0.37	0.48
Ame			—	1.73			—	0.00			—	0.11			—	0.50
Mpu				—				—				—				—
